# Morphometric Evaluation of Human Placental and Umbilical Cord for Neonatal Indices: A Cross-Sectional Study

**DOI:** 10.7759/cureus.48959

**Published:** 2023-11-17

**Authors:** Madhavi Tankala, Mohini K Rao, Susmita Senapati, Shashi Shankar Behera

**Affiliations:** 1 Department of Anatomy, Kalinga Institute of Medical Sciences (KIMS) Hospital, Bhubaneswar, IND; 2 Department of Pathology, Kar Clinic and Hospitals Pvt. Ltd., Bhubaneswar, IND; 3 Department of Obstetrics and Gynecology, Advanced Medicare & Research Institute Ltd. (AMRI) Hospitals, Bhubaneswar, IND; 4 Department of Obstetrics and Gynecology, All India Institute of Medical Sciences, Bhubaneswar, IND

**Keywords:** placental weight, newborn weight, neonatal indices, umbilical cord indices, placental indices

## Abstract

Background: In India, there is only limited data on studies related to umbilical cord and placental indices in newborn infants. The present study was undertaken to evaluate the morphometric analysis of placental and umbilical cord indices and their association with length, weight, and head size in newborns.

Materials and methods: This was a longitudinal cross-sectional study conducted on placentas and fetal measures from 245 women who gave birth during the study period. The placental variables, umbilical cord indices, and neonatal indices were measured. The association between the parameters was done using Pearson’s correlation, and p<0.05 was considered statistically significant.

Results: The placental weight (p=0.01) and placental volume (p=0.01) showed a significant positive correlation with newborn weight. The mean placental diameter was 16.32 ± 1.54 cm, and there was no significant correlation between placental diameter and infant weight (p=0.232), length (p=0.658), or head circumference (0.842). In addition, there was a significant association between placental diameter, placental volume (p=0.000), and placental weight (p=0.000). There was a significant positive correlation (p<0.05) between ponderable index and birth weight, length, and head circumference.

Conclusion: Placental weight and newborn weight were significantly associated in this study. The length of the umbilical cord was found to be related to placental weight. So, placental measurements are reliable indicators for the assessment of fetal wellbeing.

## Introduction

The two unique surfaces in the human placenta are the chorionic plate, which confronts the developing fetus, and the basal plate, which interfaces with the endometrium of the mother [1]. The placenta orchestrates fetal development processes such as breathing, nourishment, excretion, endocrine activity, and immunological support [2]. Mounting physiological and pathological conditions such as pre-eclampsia, anemia, and cigarette smoking affect the placental morphology [3-5]. The exterior surface of the umbilical cord is dull white in color, and during term it has a diameter ranging between 0.8 and 2.0 cm with a length of 55 cm, and it varies globally [6]. Placental weight alone cannot predict placental efficiency, while the feto-placental ratio is also important to measure placental functions [7].

The neonatal ponderal index (PI) is an anthropometric tool that is used to measure the growth restriction in newborns. PI is measured by dividing the infant's birth weight (grams) by their body length (cm), then multiplying the result by 100. PI accurately predicts the number of wasted newborns, irrespective of their position on the percentile lines of birth weight [8]. In the current scenario, the PI has replaced the intrauterine growth restriction (IUGR), which mainly depends on the low birth weight (LBW) for the given gestational age, as a significant predictor of infant death. So, for the evaluation of intrauterine malnutrition, LBW alone is not sufficient, and for an effective diagnosis, a ponderal index appropriate for the gestational age and body length must be considered [9].

IUGR is classified as symmetric or asymmetric, and fetal head circumference (FHC) is an important variable to measure IUGR. During symmetric IUGR, the fetus is small in all dimensions, including FHC, but in the case of asymmetric IUGR, the fetus is small with normal FHC [8,9]. LBW is also a good predictor of IUGR, but other parameters like FHC are also important for the diagnosis of IUGR [8].

Globally, perianal morbidity and mortality are common scenarios, and the burden is higher in low-resource countries. Accurate placental functions are important for fetal growth, and only limited studies are available in India related to fetal death and placental and umbilical cord abnormalities. In this backdrop, the present investigation investigated the morphometric and qualitative evaluation of the umbilical cord and human placenta and their association with neonatal variables such as fetal length, weight, and head size.

## Materials and methods

Study setting

This was a longitudinal cross-sectional study conducted on 245 women who delivered their babies at Kalinga Institute of Medical Sciences (KIMS), Bhubaneswar, Odisha, from January to July 2023.

Inclusion criteria 

Placentas from non-hypertensive, non-diabetic, and non-sickle cell disease patients with complete social and demographic information, gestational age, data in antenatal cards, and identifiable samples with attached number stickers were included in the study.

Exclusion criteria

Placentas from hypertensive, diabetic, or sickle cell disease patients were excluded, as were multiple pregnancies, unknown gestational age, the absence of antenatal care cards, HIV-seropositive women, and samples with missing or unclear number stickers.

Study methodology

The collected placentas were cleaned to remove blood stains and clots and washed thoroughly under running water. The umbilical cord was detached, leaving a 2.5-cm stump at the point of fetal implantation. In the labor ward, the placentae were examined for bleeding, with special attention paid to centrally positioned clots that might suggest placental abruption. Each specimen was given a unique number that corresponded to the neonatal index record. The mothers in the study had comprehensive data on their socio-demographic factors and gross placental measures. The various placental, umbilical cord, and neonatal variables used in this study are represented in Table 1.

**Table 1:** Different placental, umbilical cord, and neonatal indices were used in the study

Placental indices	Umbilical cord indices	Neonatal indices
Placental weight	Umbilical cord diameter	Birth weight
Placental thickness	Umbilical cord length	Head circumference
Placental diameter	Umbilical cord insertion	Body length
Placental shape	Cord centrality index	Abdominal circumference
Chorionic plate area	Umbilical cord vessel number	Ponderal index
Placental completeness determination	Umbilical cord area	
Circumvallate placenta determination	Umbilical cord volume	
Amnion nodosum		

Placental variables

Placental Weight

The placentae (comprising placental membranes and umbilical cords) were weighed with the help of a mechanical kitchen scale with a range of 0-5000g (Zhongshan Camry Model: KCH).

Placental Thickness

Placental thickness was determined by the toothpick technique. The placentae were pierced at nine separate sites along two perpendicular planes. The collected measures were transferred to a transparent ruler calibrated in centimeters (Helix China Inc., 30 cm/12 inches), and the average values were calculated.

Placental Diameter

A Dritz C150 fiber glass measuring tape was used to measure the placental diameter. The mean value of four distinct angles in each placenta was measured. This method was chosen since most placentae had a discoid or ovoid form upon physical inspection, necessitating numerous measurements.

Placental Shape

The placenta was classified as round, irregular, oval, and bilobate. The eccentricity of an ellipse or oval was used to check the placental shape. Eccentricity index, the ratio between foci and length of the main axis, were calculated. A suitable eccentricity index is between 0 and 1, with 0 indicating a circular form and values closer to 1 indicating an elliptical shape.

Chorionic Plate Area (Square cm)

An ellipse’s area was calculated using the major as well as the minor diameter of the chorionic disc. The formula for this computation was A = π x (dL/2) x (dS/2), with a representing the area of the chorionic disc, with dL representing the placental diameter (major) and dS representing the placental diameter (minor) [10].

Placental Completeness Determination

Prior to fixation, an evaluation was performed at the labor ward to determine the completeness of the placenta. The cleaned placenta was thoroughly scrutinized for any damaged tissue. The surface on the maternal side was examined for the presence of all cotyledons. The existence of vessels across the margins suggests that a full placental lobe, such as a succenturiate lobe, may be retained.

Circumvallate Placenta Determination

The thick membranous ring on the placental fetal surface was taken into account for the measurement of the circumvallate placenta. A narrow ring of membrane tissue represents circummarginate of the placenta.

Amnion Nodosum

These were described as many tiny, hard nodules in white or yellow color on the placental fetal surface.

Umbilical cord indices

Umbilical Cord Diameter and Length

A conventional non-elastic tape measure was used to measure the whole length, beginning at the fetal end and terminating at the site of entry into the placenta. Umbilical cord lengths were classified into three categories: short if they were less than 40 cm, normal if they were between 40 and 70 cm, and long if they were greater than 70 cm.

Umbilical Cord Insertion

They were classified as central, marginal, eccentric, or velamentous.

Cord Centrality Index

It was measured as a ratio of the distance between the insertion of the umbilical cord and the margin of the chorionic plate. A lower CI value indicates that the insertion of the umbilical cord is closer to the center of the placenta, whereas a higher CI value indicates that cord insertion is further away.

Umbilical Cord Vessel Number

**Figure 1:**
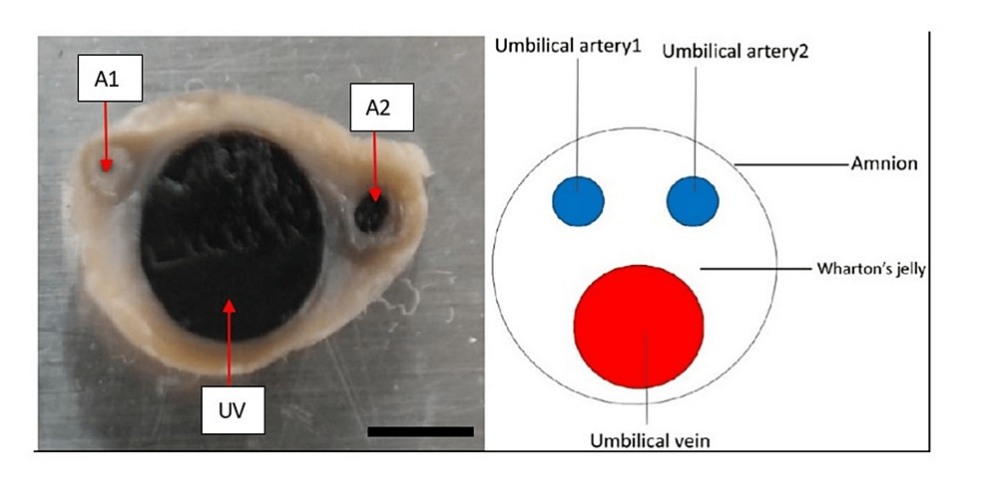
Schematic diagram showing the vessels in umbilical cord

The number of umbilical cord vessels was determined by slicing the cord at both ends and comparing the number of vessels observed at each end. The entire vessel area was deducted from the area of the umbilical cord to compute the Wharton jelly’s surface cross-sectional area (Figure 1).

Neonatal indices

All newborn measurements were measured within 24 hours of birth. The Dritz C150 fiber glass standard tape was used to measure head size, body length, and circumference of the abdomen. PI was measured by dividing the infant's birth weight (grams) by their body length (cm), then multiplying the result by 100. The head circumference to abdominal ratio was obtained by dividing the head circumference (cm) by the abdomen circumference (cm).

Ethical issues

The study was approved by the Institutional Ethical Clearance Committee of Kalinga Institute of Medical Sciences (KIIT/KIMS/421/2022).

Statistical analysis

The continuous variables such as neonate, placenta, umbilical cord, and vessels were represented as mean and SD (standard deviation). The association between different placental and umbilical cord measurements and newborn anthropometric measures was evaluated using Pearson’s correlation analysis. A p-value of <0.05 was considered significant. Graph pad prism V7 was used for the analysis.

## Results

Among the 245 neonates, 129 (52.65%) were males, and 116 (47.35%) were females. The descriptive statistics of neonatal, placental, and umbilical cord indices are shown in Table 2.

**Table 2:** Neonatal, placental, and umbilical cord indices among the neonates (N = 236) Data are expressed as Mean ± SD, Standard Error, Range with minimum and maximum limits, Coefficient of variation, SD: Standard Deviation, SE: Standard Error, CoV: Coefficient of Variation. BW: Birth Weight, BL: Body Length, HC: Head Circumference, PI: Ponderal Index, PD: Placental Diameter, PW: Placental Weight, PT: Placental Thickness, PA: Placental Area, PV: Placenta Volume, UCL: Umbilical Cord Length, UCD: Umbilical Cord Diameter, UCA: Umbilical Cord Area, UCV: Umbilical Cord Volume.

Variables		Mean ± SD	Range	CoV
Neonatal indices	BW (kg)	3.12 ± 0.45	1.21 to 4.54	15.76
	BL (cm)	50.45 ± 3.12	32.0 to 62.0	6.78
	HC (cm)	33.12 ± 1.74	25.0 to 48.0	5.45
	PI	2.12 ± 0.42	0.8 to 8.2	24.12
Placental indices	PD (cm)	16.32 ± 1.54	10.87 to 24	10.34
	PW (g)	576.65 ± 119.56	141 to 1024	22.76
	PT (cm)	2.12 ± 0.25	1.6 to 3.24	22.15
	PA (cm2)	242.28 ± 49.12	106.12 to 416.24	21.24
	PV (cm3)	482.85 ± 132.18	201.05 to 934.76	27.76
Umbilical cord indices	UCL (cm)	42.28 ± 9.12	15 to 76.87	26.43
	UCD (cm)	1.32 ± 0.09	0.45 to 2.32	16.12
	UCA (cm2)	94.12 ± 24.12	25.28 to 412.76	50.12
	UCV (cm3)	47.12 ± 12.45	9.12 to 154.12	55.25

Among the 245 umbilical cords investigated, 125 (51.02%) were short, 112 (45.71%) were normal, and eight (3.26%) were long. The mean diameter of the A1 and A2 umbilical cord arteries was 0.16 ± 0.02 cm and 0.22 ± 0.04 cm, respectively. The mean area and volume of umbilical cord artery A1 were 1.52 ± 0.21 mm2 and 0.82 cm3 ± 0.09, respectively. The mean area and volume of umbilical cord artery A2 were 2.38 ± 0.12 cm2 and 1.20 cm3 ± 0.01 cm3, respectively (Table 3).

**Table 3:** Summary of the umbilical vein with Wharton Jelly’s morphometry Data are expressed as Mean ± SD, Standard Error, Range with minimum and maximum limits, Coefficient of variation, SD: Standard Deviation, SE: Standard Error, CoV: Coefficient of Variation, UCVD: Umbilical Cord Vein Diameter, UCVA: Umbilical Cord Vein Area, UCVV: Umbilical Cord Vein Volume, AWJ: Area of Wharton’s Jelly, VWJ: Volume of Wharton’s Jelly.

Variables	Mean ± SD	Range	CoV
UCVD (cm)	0.42 ± 0.01	0.17 to 0.65	39.23
UCVA (cm2)	12.34 ± 2.54	3.12 to 42.54	87.12
UCVV (cm3)	4.87 ± 0.98	7.76 to 76.32	101.54
AWJ (cm2)	82.12 ± 21.43	20.12 to 154.29	52.12
VWJ (cm3)	42.34 ± 12.32	7.12 -78.34	56.86

For placentas weighing about 350-750 grams, the prevalence of central insertions was 44 (17.96%), eccentric insertions were 150 (61.22%), peripheral insertions were 30 (12.24%), and there were no cases of velamentous insertions. For placentas weighing >750g, the frequency of central insertions was five (2%), eccentric insertions were 12 (49%), marginal insertions were three (1.22%), and velamentous insertions were one (0.41%), respectively.

With respect to central umbilical cord insertions, the frequency was higher in females as compared to males (28 [11.42%] vs. 24 [9.79%]). Likewise, the marginal insertion was higher in females as compared to males (22 [8.98%] vs. 16 [6.53%]). In contrast, the frequency of eccentric insertions was higher in males as compared to females (81 [33.06%] vs. 74 [30.20%]). Meanwhile, velamentous cord insertion was observed in one female neonate, accounting for 0.41%. The distribution of placentae and birth weight among neonates is shown in Table 4.

**Table 4:** The distribution of placentae and birth weights among neonates

Birth weight (kilograms)	Gender		Placentae weight (grams)	Gender	
	Male	Girl		Boy	Girl
<2.5	12	10	< 350	6	3
2.5 to 4.0	106	101	350 to 750	114	103
>4.0	11	5	> 750	9	10

The birth weight of the female neonates ranged from 1.31 to 4.25 kg, and the placental weight ranged from 260 to 1060 g, with a mean value of 625g. In male neonates, the birth weight ranged from 1.35 to 4.76 kg, and the placental weight ranged from 180 to 980 g, with a mean value of 542g. The feto-placental ratio among the female and male neonates was found to be 5.45 and 5.78, respectively. The placental shape was 73 (29.79%) round, 133 (54.28%) irregular, 33 (13.47%) oval, and four (1.63%) bilobate, respectively.

In this study, there was a significant association between weight and volume of placenta and neonatal birth weight. Linear associations were found with placental and birth weights (R2=0.034, p=0.01) and with placental volume and birth weight (R2=0.072, p=0.01). Birth weight showed a significant correlation with body length and head circumference. Head circumference and weight (R2=0.085, p=0.001) and body length and weight (R2=0.387, p=0.001) showed significant linear correlations.

**Figure 2:**
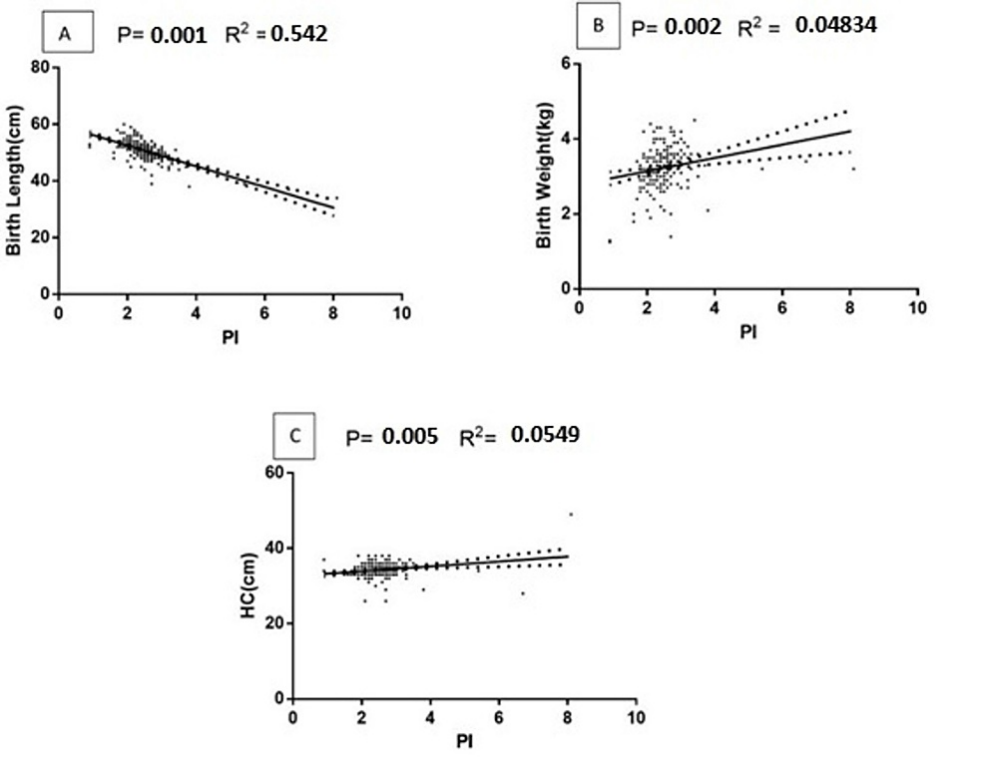
Relation between the ponderal index and neonatal indices

The ponderal index showed a significant correlation with birth length, birth weight, and head size. However, birth length (p=0.001, R2=0.542) showed inverse association with ponderal index, although birth weight (p=0.002, and R2=0.04834) and head circumference (p=0.005, R2=0.0549) displayed positive association with ponderal index. The results are shown in Figures 2A-2C.

Birth length (p=0.001), head circumference (p=0.008), and placental weight (p=0.005) showed significant positive associations with birth weight. Further, birth length showed a significant association with placental shape (R2=0.239; p=0.018). Meanwhile, head circumference showed no significant correlation with any of the placental indices. Placental thickness showed a significant correlation with the insertion of the umbilical cord (p=0.004) and placental weight (p=0.000). The results are shown in Table 5.

**Table 5:** Summary of correlation analysis between neonatal indices and placental indices **. Correlation is significant at the 0.01 level: *. Correlation is significant at the 0.05 level (2-tailed). BW: birth weight, BL: birth length, HC: head circumference, PS: placenta shape, UCI: umbilical cord insertion, PW: placenta weight, PT: placenta thickness, PD: placenta diameter, PV: placenta volume.

		BW	BL	HC	PS	UCI	PW	PT	PD
BW	Pearson Correlation	1							
	Sig. (2-tailed)								
BL	Pearson Correlation	0.542**	1						
	Sig. (2-tailed)	0.001							
HC	Pearson Correlation	0.369**	0.122	1					
	Sig. (2-tailed)	0.008	0.062						
PS	Pearson Correlation	-0.0279	0.239*	0.035	1				
	Sig. (2-tailed)	0.786	0.018	0.625					
UCI	Pearson Correlation	0.052	0.064	-0.032	0.134	1			
	Sig. (2-tailed)	0.365	0.387	0.475	0.142				
PW	Pearson Correlation	0.272*	0.085	-0.026	0.043	0.095	1		
	Sig. (2-tailed)	0.005	0.156	0.728	0.521	0.157			
PT	Pearson Correlation	0.062	-0.024	0.243	-0.038	0.294**	0.487**	1	
	Sig. (2-tailed)	0.287	0.652	0.072	0.652	0.003	0.000		
PD	Pearson Correlation	0.064	0.032	0.045	0.142*	-0.046	0.384**	-0.065	1
	Sig. (2-tailed)	0.232	0.658	0.842	0.025	0.535	0.000	0.215	
PV	Pearson Correlation	0.055	0.027	0.062	0.084	0.037	0.326**	0.345**	0.856**
	Sig. (2-tailed)	0.165	0.542	0.429	0.252	0.548	0.01	0.000	0.000

Meanwhile, there was a weak positive correlation between ponderal index and birth weight (R2=0.322; p=0.03) and head circumference (R2=0.365; p=0.02) and a strong negative correlation between ponderal index and birth length (R2=-0.677; p=0.001), respectively.

## Discussion

This study presents an extensive examination of gross morphometric parameters in human placentas and umbilical cords. Our research endeavors to provide valuable insights into the relationship between neonatal indices and placental/umbilical cord characteristics, contributing to a broader understanding of fetal development and its implications for maternal and neonatal healthcare. The study encompassed a cohort of 245 neonates, comprising 52.65% males and 47.35% females. A detailed exploration of neonatal indices, including birth weight (BW), body length (BL), head circumference (HC), and ponderal index (PI), was conducted. These indices are critical for assessing the physical development of neonates. The mean placental weight in our study was determined to be 578.81 grams. The observed patterns in these indices align with prior research conducted in India, providing a basis for comparison and reinforcing the universality of these fundamental measures [11]. This figure did not exhibit any statistically significant deviation when compared to similar studies conducted in Israel [12] and Western Europe [13]. Nevertheless, it was found to be somewhat lower than the figures reported in Sokoto, Nigeria [14].

Drawing upon prior research conducted by Sanin et al. [15], which uncovered a 1.98-gram increase in fetal weight for every gram increase in placental weight, our study elucidates a comparable increase of 2 grams in birth weight for every gram increase in placental weight. This affirmative relationship between placental and newborn weights underscores the potential utility of placental weight as a predictor of neonatal weight, reflecting the influence of various maternal and environmental variables. These factors encompass maternal attributes such as height, hemoglobin levels, and altitude, as well as paternal and genetic factors from both parents [16]. Notably, our investigation did not reveal any significant relationships between placental diameter and infant weight, head circumference, or length. However, significant correlations were observed between placental diameter and the weight and volume of the placenta. The measurement of placental diameter can offer valuable insights into its size, thereby indirectly indicating the fetal-placental ratio. This finding aligns with previous research by Borton [17] and Ohagwu et al. [18], who reported placental diameters at term within the range of 15-25 cm. In comparison, Yetter [19] observed a mean diameter of approximately 22 cm. Our study's mean placental diameter was larger than Yetter's [19] findings but remained consistent with the limits reported by Borton [17] and Ohagwu et al. [18].

The average placental thickness in our research was measured at 2.04 cm, with a significant relationship identified between thickness and placental weight. In terms of umbilical cord characteristics, we observed variations in cord length, ranging from 16 to 80.5 cm, with an average length of 41.74 cm. These measurements revealed that 50.85% of cords were classified as short, 46.61% as normal, and 2.54% as long. Importantly, a significant association was established between umbilical cord length and placental weight. Our study further delved into the dimensions of the umbilical cord, with the average diameter found to be 1.19 cm, ranging from 0.65 to 2.0 cm. These findings closely parallel those of Collins, who reported a diameter of 1.2 cm in their study. Nevertheless, notable disparities were identified when comparing our data from Ghanaian samples (1.19 cm) with data from Sudan and Nigeria (1.2 cm and 1.4 cm, respectively) [20,21].

Additionally, our investigation assessed the dimensions of umbilical artery segments, revealing a mean diameter of 0.14 cm for A1 and 0.18 cm for A2, while the umbilical vein displayed a diameter of 0.38 cm. Consistent with established anatomy, the human umbilical cord typically consists of two helical arteries and a straight vein [22]. It is worth noting that the issue of segmental decrease in artery diameter and the potential non-circular shape of the vessel post-delivery can be effectively addressed by utilizing volume and area measurements of the umbilical artery. Moreover, volume measurements provide a comprehensive perspective of the entire arterial space, facilitating the estimation of blood flow velocity. This aligns with the findings of Togni et al., who reported a strong correlation between gestational age and umbilical artery area [23]. In terms of umbilical cord insertion, our study revealed a distribution of 13.14% marginal, 0.42% velamentous, 19.49% central, and 66.95% eccentric insertions, resulting in an 86.44% combined central/eccentric insertion rate. This combined percentage closely aligns with the findings of Kouyoumdjian [24], who reported that nearly nine-tenths of cord insertions were either eccentric or central.

Furthermore, our investigation yielded data regarding average birth weight (3.24 kg), head circumference (34.27 cm), and neonatal length (50.64 cm). Notably, a higher head circumference was positively correlated with a greater neonatal weight, rendering it an indirect measure of neonatal weight. Our study's mean head circumference of 34.27 cm was consistent with Eregie's measurements in Benin-City, Nigeria (34.2 cm) [25] and Jos (34.49 cm) [26]. Additionally, the mean newborn length in our study was 48.8 cm, like the findings of Lo et al. [27]. Incorporating the findings of Raghunath G et al. [11], Salmani D et al. [28], Chakraborty S et al. [29], and Sirpurkar M et al. [30] into the broader context of placental and neonatal research, it becomes evident that our study contributes valuable insights into the intricate relationships between placental and neonatal parameters. These findings enhance our understanding of the multifaceted factors influencing fetal development and birth outcomes.

Raghunath G. et al. conducted a study in Tamil Nadu, providing insights into placental shape, dimensions, and various associated factors. Their findings underscore the relevance of placental characteristics to fetal development [11]. Salmani D et al. conducted a study in Mahbubnagar, Andhra Pradesh, highlighting differences in placental weight and dimensions between the study and control groups. They also emphasized the significant impact of hypertensive conditions on neonatal birth weight and placental morphology [28]. Chakraborty S. et al. conducted research in Kolkata, elucidating placental morphometric parameters in normal and diabetic pregnancies. Their findings underscore the predictive value of neonatal birth weight and maternal factors in relation to placental characteristics [29]. Sirpurkar M et al. conducted a study in Bhopal, shedding light on variations in placental weight among different pregnancy complications. Their findings highlight the potential influence of various medical conditions on neonatal outcomes [30].

Limitations: The limitations of this study include its small sample size, cross-sectional study design, and measurement inaccuracies. Addressing these concerns is crucial for ensuring the study's validity. It's important to critically assess the methodology, analysis, and potential biases to fully understand the scope and implications of the research.

## Conclusions

The current study found a link between newborn and placental weights, along with placental volume, but no association between neonatal weight and placental diameter or thickness. Similarly, umbilical cord length was linked to placental weight but not to newborn weight.
